# Soft and Deformable Sensors Based on Liquid Metals

**DOI:** 10.3390/s19194250

**Published:** 2019-09-30

**Authors:** Taeyeong Kim, Dong-min Kim, Bong Jae Lee, Jungchul Lee

**Affiliations:** 1 Department of Mechanical Engineering, Korea Advanced Institute of Science and Technology, Daejeon 34141, Koreaduracell@kaist.ac.kr (D.-m.K.); 2 Center for Extreme Thermal Physics and Manufacturing, Korea Advanced Institute of Science and Technology, Daejeon 34141, Korea

**Keywords:** liquid metals, deformable sensors, flexible electronics, soft sensors

## Abstract

Liquid metals are one of the most interesting and promising materials due to their electrical, fluidic, and thermophysical properties. With the aid of their exceptional deformable natures, liquid metals are now considered to be electrically conductive materials for sensors and actuators, major constituent transducers in soft robotics, that can experience and withstand significant levels of mechanical deformation. For the upcoming era of wearable electronics and soft robotics, we would like to offer an up-to-date overview of liquid metal-based soft (thus significantly deformable) sensors mainly but not limited to researchers in relevant fields. This paper will thoroughly highlight and critically review recent literature on design, fabrication, characterization, and application of liquid metal devices and suggest scientific and engineering routes towards liquid metal sensing devices of tomorrow.

## 1. Introduction

Historically, metals have played a key role in the civilization of mankind and the industrial revolution. While solid-state metals have been perfect and ideal candidates for major engineering/scientific disciplines and industries, they are not well suited for applications and products requiring decent electrical properties under relatively large mechanical deformation. For example, solid-state metallic nanowires show irreversible conductivity recovery when they are relaxed from large strains and show hysteresis in repeated cycles [1,2,3,4]. While the meander [5], serpentine [6,7,8], or kirigami [9] structures, which are made of thin metallic conductors, improve the strain range up to 300% [8], they have a limited operating range and cannot withstand more extreme strain [10]. Since form factors of computing and communication devices are now rapidly changing from portable to wearable, it is very important to look for and study materials ideal for such a new trend.

Liquid metals, liquid at or near room temperature, may be ideal candidates for devices subjected to large mechanical strains considering their electrically conductive and freely deformable natures. While one of the elemental liquid metals, mercury, has been phased out due to its toxicity, gallium-based alloys are now gaining attention for the development of new flexible and stretchable devices. Among them, soft and deformable sensors are certainly mainstream. To make soft and deformable sensors with liquid metals, they need to be properly patterned on and encapsulated or passivated with materials that also withstand significant degrees of mechanical deformation.

Prerequisites for the successful development of liquid metal-based soft sensors include knowledge and knowhow regarding fundamental properties and characteristics of liquid metals and various patterning methods for liquid metals. In addition, not to reinvent the wheel, we should be well aware of liquid metal devices and applications already demonstrated. To provide a systematic overview and guideline for future development, this paper will thoroughly review recent representative works on fundamental characteristic behaviors of liquid metals (mostly gallium-based alloys), various patterning techniques for liquid metals, and characterizations and applications of liquid metal-based devices.

## 2. Fundamentals of Liquid Metals

Liquid metals refer to materials with melting point lower than room temperature thus they remain liquid state at room temperature. With the aid of negligible vapor pressure [11] and low toxicity, gallium-based alloys become the most popular liquid metals. In addition, gallium-based alloys exhibit relatively higher electrical and thermal conductivities than a traditional liquid metal, mercury [12,13]. Therefore, gallium-based alloys are more suited for highly deformable sensor applications [14,15,16]. Representative gallium-based alloys are EGaIn (Ga:In = 75.5:24.5 in weight ratio) and galinstan (Ga:In:Sn = 68.5:21.5:10 in weight ratio). Hereafter, liquid metals exclusively refer to gallium-based alloys. In this section, fundamental properties of liquid metals crucial for the development of deformable sensors will be discussed. Electrical and thermal conductivities, surface oxide and rheological properties will be covered.

### 2.1. Electrical Conductivity

Regardless of phase of conductive materials, electrical conductivity is one of the most important characteristics for current carrying devices. While electrical conductivity measurement for solid-state materials is typically based on the straightforward four-probe potentiometric method (i.e., applying electrical current to two terminals of a specimen with known dimensions and measuring voltage), special preparation, consideration, and modification are required for liquid-state materials. For example, liquid metals exhibiting fluidic natures are filled in a precision glass capillary with uniform cross-section and evacuated to remove trapped gas bubbles. Then, electrodes are connected to two end terminals to apply current and measure induced voltage [17]. Except the capillary filling and degassing, the remaining procedures are equivalent to those for solid-state materials. The relatively simple capillary method is mainly used for mercury but can be problematic if electrodes chemically react with liquid metals under test. To overcome this drawback, a contactless method to measure electrical conductivity was developed. When circulating eddy currents are induced in a moving sample, damping torque is produced and it depends on electrical conductivity. Therefore, by measuring the damping torque, electrical conductivity of a liquid metal can be obtained [18].

**Figure 1:**
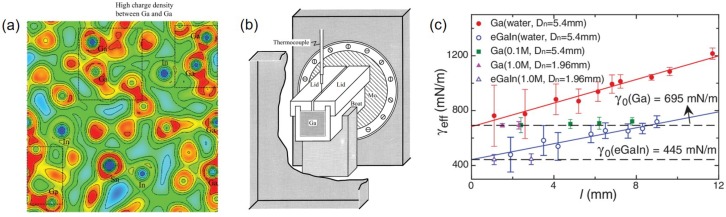
(**a**) A contour of charge density for liquid EGaInSn at 310 K. The color scale is from 0 (blue) to 0.038 (red) $e_{c}{Å}^{-3}$. Reprinted from [19], with the permission of AIP Publishing. (**b**) A schematic of the experimental apparatus for measuring thermal conductivity of gallium. Reproduced with permission from [13]. (**c**) Measured effective surface tension vs. drop diameter, *l*, for gallium and EGaIn in water or HCl baths with different concentrations. Reprinted from [25], with the permission of AIP Publishing.

At room temperature, gallium, EGaIn and galinstan have electrical conductivities of $3.4\times 10^{6}$ S·m^−1^, $3.3\times 10^{6}$ S·m^−1^, and $3.1\times 10^{6}$ S·m^−1^, respectively [17,19]. Similar electrical conductivities for gallium, EGaIn and galinstan may be attributed for the fact that most free electrons exist around gallium atoms (Figure 1a). Electrical conductivities of gallium or gallium-based alloys are one order of magnitude lower than those of decent solid conductors such as silver ($6.3\times 10^{7}$ S·m^−1^) or copper ($5.96\times 10^{7}$ S·m^−1^) [20] at room temperature but one order of magnitude higher than that of a famous thus popularly used conductive polymer, PEDOT:PSS ($3.1\times 10^{5}$ S·m^−1^) [21]. If both electrical conductivity and strain limit are considered together, liquid metals are ideal candidates for deformable sensors or electronics.

### 2.2. Thermal Conductivity

For thermal conductivity measurement, temperature gradient is necessary to be developed within a relatively long and slender specimen. Since heat (thermal current) flow is analogous to electrical current flow, thermal conductivity measurement is certainly akin to electrical conductivity measurement. Similar to the capillary method [17], a liquid metal is contained inside a boat of rectangular cross-section of which both ends are fixed with end plates (Figure 1b). When one end plate is heated to higher temperature and the other end plate is cooled to lower temperature, temperature gradient is developed within the filled liquid metal. There is an open and narrow slot on the top lid of the liquid metal container where a thermocouple attached to a linear manipulator can access the liquid metal and measure its temperature along the direction parallel to the temperature gradient. Once heat loss from the boat to surrounding is considered, thermal conductivity can be extracted using the heat conduction equation [13]. Alternatively, thermal conductivity of a liquid metal can be measured by the transient hot wire method, a representative method for measuring thermal conductivity of a fluid [22].

The thermal conductivities of gallium, EGaIn and galinstan are 30.54 W·m^−1^·K^−1^, 26.43 WW·m^−1^·K^−1^ and 25.41 W·m^−1^·K^−1^ at room temperature, respectively [19]. These values are one order of magnitude lower than those of good solid heat conductors such as silver (429 W·m^−1^·K^−1^) and copper (401 W·m^−1^·K^−1^) [23] but two orders of magnitude higher than that of the conductive PEDOT:PSS polymer (0.17 W·m^−1^·K^−1^) [24]. Such trends are qualitatively similar to relative magnitude comparison of electrical conductivities of solid metals, liquid metals, and conductive polymer. Since liquid metals exhibit relatively high thermal conductivity among flexible electronic materials, they can aid thermal management of electronic devices that is important for wearable electronics.

### 2.3. Surface Oxide

Except noble metals and corrosion-resistant alloys, metals tend to be oxidized when their surfaces are exposed to air, water, or acids. Most generally in air, ionic chemical reaction occurs where electrons move from the metal to the oxygen molecules. As a result, negative oxygen ions generated react the metal and create surface oxide. Gallium or its alloy are not exception. When they are exposed to oxygen, even at very low concentration of ∼1 ppm, surface oxide forms. Surface oxides are gallium oxides (${\mathrm{Ga}}_{2}O_{3}$) since gallium is oxidized faster than indium or tin [26,27]. Gallium oxide formation is self-stopping that can be explained by the Cabrera and Mott model [28,29]. Mott potential developing due to the energy level difference between liquid metal and surface oxide effectively reduces the energy barrier to enable the passage of cations and anions into the surface oxide. Increment of the oxide thickness reduces the effect of the Mott potential and the oxidation process stops at a certain thickness [30].

There have been several attempts to quantify thickness of surface oxides around gallium or its alloys. One way is to separate the surface oxide from the liquid metal and measure its thickness afterwards. By making contact the liquid metal surface with a solid substrate, the surface oxide can be exclusively separated with the aid of van der Waals force. Since there is no macroscopic force between the inner bulk liquid metal and the surface oxides, the surface oxide can be separated cleanly by contacting with a suitable substrate such as silicon [31]. In addition, by manually sucking and removing the liquid metal droplets and wiping them with a cured polydimethylsiloxane (PDMS) substrate, the liquid metal can be removed by the same principle as mentioned above [32]. Pressurized air injection can also be employed to separate surface oxides from liquid metal. Oxides form rapidly in the air bubble and they are separated in the aqueous solution [31]. Once surface oxides are separated, atomic force microscopy (AFM) is applied to measure their thicknesses. AFM reveals that oxides separated by the van der Waals exfoliation are ∼2.8 nm [31], oxides separated by the PDMS wiping are 2.7∼5.9 nm thick [32], and oxides separated by the pressurized air injection are ∼5.2 nm [31]. It is believed that variation in gallium oxide thicknesses is ascribed to the oxygen concentration during oxidation, oxidation time, and the amount of liquid metal residues after separation.

Interestingly, the presence of the surface oxide is useful for mechanical integrity as well as electrical functionality. When the surface oxide is removed upon exposure to hydrochloric acid (HCl), the contact angle between liquid metal and substrate significantly increases and dewetting occurs [25]. Therefore, without the surface oxides, liquid metal patterns cannot maintain adhesion to substrate. In addition, surface oxides can also be applied to switching devices such as field-effect transistors (FETs) by making use of their semi-conducting natures [32].

### 2.4. Rheological Properties

Rheological properties of liquid metals are somewhat unique and important because they are liquid surrounded by a thin solid shell. Although both thickness and total volume of the surface oxide are negligible small compared to amount of the enclosed liquid metal, its effect can be significant. When a liquid metal is introduced into a microchannel, its surface tension can be calculated from the contact angle with the microchannel wall and the width and height dimension of the microchannel using the Young-Laplace equation [26]. Later, the pendant drop experiment, a standard surface tension measurement technique, is applied to liquid metals. After immersing a liquid metal droplet discharged from a nozzle in water with or without HCl added, surface tension is extracted by fitting the equilibrium shape of the liquid metal droplet using the Laplace equation. Due to the absence of the typical liquid-gas interface, the extracted surface tension is called effective surface tension. In general, gallium exhibits surface tension higher than EGaIn. As the droplet diameter increases in pure water (i.e., without adding HCl so the surface oxide remains intact), surface tensions of both gallium and EGaIn increase due to the increased surface of the oxide layer. However, when HCl is added, surface tensions of both gallium and EGaIn become independent of the droplet diameter. In case of gallium, surface tension values are also independent of the HCl concentration (Figure 1c). Since addition of HCl removes the surface oxide, the diameter dependence of surface tension exclusively observed with the surface oxide cannot be seen any further [25]. When the surface oxide is removed, surface tensions of gallium and EGaIn are measured to be ∼695 mN·m^−1^ and ∼445 mN·m^−1^ [26]. In comparison, surface tension of EGaIn is measured to be ∼630 mN·m^−1^ in air at room temperature [25]. This is higher than surface tensions of water (∼73 mN·m^−1^) and mercury (∼465 mN·m^−1^) at room temperature [33,34]. Surface tension can also be controlled by varying the interfacial tension between liquid metal and solution using electrocapillarity. For example, the liquid metal in sodium hydroxide (NaOH) forms an electrical double layer (EDL) and the voltage drop across the EDL changes the interfacial tension. This in turn changes the surface tension of the liquid metal. This idea can be used to drive and steer liquid metals within microchannels with the surface tension gradient that induces the Marangoni flow [35].

Viscosity of liquid metals can be measured using rheometers that measure the dynamic viscosity of conventional fluids. However, liquid metals have lower viscosity than conventional fluids, so they were calibrated with a known Newtonian fluid that exhibits the same scaling relationship between dimensionless stress and Reynolds number [25]. Gallium and EGaIn have kinematic viscosities of $3.347\times 10^{-7}$ m^2^·s^−1^ and $3.213\times 10^{-7}$ m^2^·s^−1^, respectively [34]. These values are lower than kinematic viscosities of water and mercury that are $1.003\times 10^{-6}$ m^2^·s^−1^ and $1.35\times 10^{-6}$ m^2^·s^−1^, respectively, at room temperature [36,37].

Without the surface oxide, liquid metals are shaped spherically to minimize their surface energy. However, the surface oxide tends to change the shape of liquid metals because the oxide has significant effect on rheological properties of liquid metals [38]. As the oxide increases, viscosity, mechanical strength, and interfacial tension of liquid metals increase in general [39,40]. Such properties induced by the surface oxide can result in non-spherical shape of liquid metals despite their high surface tension. Therefore, liquid metals can be patterned to have non-spherical shapes with the aid of the surface oxide. When the surface stress is ∼0.5 N·m^−1^ or more and strain is 0.01 or more, they behave like liquids [26,41]. This means that liquid metals behave as viscous fluids when they are deformed. Once the applied mechanical deformation is restored, liquid metals behave as elastic materials due to the surface oxide. Therefore, liquid metals are basically viscoelastic.

## 3. Various Patterning Methods for Liquid Metals

To make use of promising liquid metals for scientific and engineering applications, it is prerequisite to pattern them properly on a target substrate. Metallization in conventional MEMS fabrication processes, either lift-off or etching in conjunction with photolithography, cannot be directly applied to liquid metals. In addition, liquid metals are usually used with soft polymers rather than hard silicon or glass wafers. Therefore, new patterning methods are necessary for liquid metals. In this section, various patterning method for liquid metals including lithography-assisted patterning, either additive or subtractive approaches, injection into microfabricated channels, and nanoparticle sintering will be discussed.

### 3.1. Lithography-Assisted Patterning

#### 3.1.1. Lift-Off

**Figure 2:**
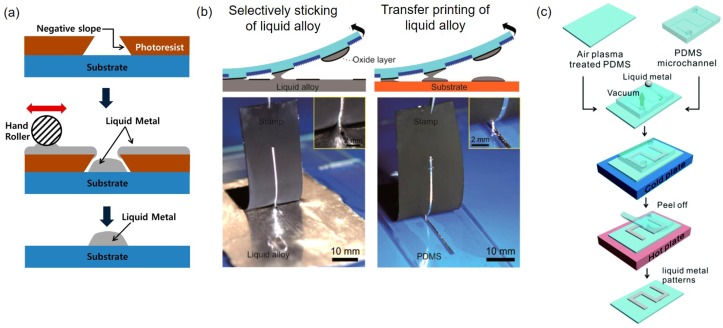
(**a**) Liquid metal lift-off process. Photoresist is photo-patterned on a substrate and liquid metal is applied using a roller. Lift-off process removes the photoresist along with liquid metal overlaid and leaves the liquid metal pattern on the substrate. Reprinted with permission from [42]. Copyright (2016) American Chemical Society. (**b**) Conceptual schematics and corresponding photographs of modified wettability mediated patterning of liquid metals. A surface-modified mold enables selective wetting of liquid metals and can be used as a stamp. Reprinted with permission from [43]. Copyright (2019) American Chemical Society. (**c**) Liquid metal filled microchannels are used as molds for contact patterning. Reproduced from Ref. [44] with permission from the Royal Society of Chemistry.

The lift-off process that relies on lithography of photoresist, deposition of metal, and removal of the patterned photoresist and overlaid metal can also be applicable to liquid metals. After lithography of photoresist (approximately 10 μm thick), EGaIn can be placed on the substrate with the patterned photoresist. There is undercut of 5∼15 μm that prevents EGaIn wetting on the side walls of the photoresist pattern [42]. By using a hand roller that applies a normal force, thickness of the EGaIn can be made uniform (Figure 2a). After the lift-off process, EGaIn patterns with the width as small as 20 μm and the height of 10 μm are obtained. While the pattern width can be easily increased by photomask design, the 10 μm height can be well maintained. The height of EGaIn patterns can be also further decreased by controlling the thickness of the photoresist. Since photolithography can be precisely performed on top of solid organic or inorganic patterns prepared previously, liquid metals can be integrated with solid-state microdevices. Overall, the liquid metal patterning via lift-off is promising. However, cleanroom access is necessary to perform photolithography and excessive liquid metal materials can be consumed unless extra materials applied are properly collected.

#### 3.1.2. Stamp

Liquid metal patterns can be made by using a stamp prepared by lithography [45]. This method relies on surface morphology tuning and controls the patterning by manipulating the wettability of liquid metals. For the stamp surface modification, one method is to reduce the effective surface contact area by using ultraviolet (UV) laser. When the stamp surface is UV-laser treated, a liquid alloy-phobic area is generated that in turn causes the pattern to be transferred onto a more liquid alloy-philic substrate (Figure 2b) [43]. Alternatively, surface properties can be changed by chemical methods. For example, toluene treatment increases the hydrophobicity of the PDMS surface that minimizes the deposition of EGaIn thus allows selective patterning [46]. The lateral patterning resolution determined by the width of the stamp is ∼2 μm while the pattern height can be made smaller than the pattern width (∼1.6 μm) [43,46]. Although the stamp method can deposit thin liquid metal films smoothly and uniformly, residues may be accumulated on the stamp surface if the stamp is used repeatedly. Accumulated residues significantly deteriorate the patterning quality.

#### 3.1.3. Mold

A microchannel made by soft lithography can be used as a mold for liquid metal patterning (Figure 2c). A PDMS layer with a microchannel molded is bonded to another bare PDMS layer with UV treated and then EGaIn is filled into the microchannel by applying vacuum. After filling the EGaIn, the whole device is cooled on a cold plate at −10 °C. Then, the device is placed on a hot plate at 60 °C and the PDMS layer with the microchannel is peeled off. Finally, the EGaIn pattern remains on the plasma treated bare PDMS layer since the surface oxide of the liquid metal results in better adhesion with plasma treated PDMS [44]. While the width of the EGaIn pattern is about the same as the width of the microchannel, the pattern height is about 10% larger than the height of the microchannel. This is attributed to the volume expansion during solid-to-liquid phase change that is more limited in the horizontal direction. The cross-sectional area can be controlled by adjusting the width and height of the microchannel but the master mold for the PDMS microchannel requires cleanroom fabrication.

### 3.2. Additive Approaches

#### 3.2.1. Direct Printing

Direct printing extrudes a liquid metal through a dispensing tip, usually a syringe needle, onto a target substrate in close proximity. In general, the dispensing tip or the substrate is attached to motorized stages that can move as programmed. In contrast to lithography-assisted patterning, the direct printing does not require any preparation effort in cleanroom. One simple way of liquid metal direct printing is the liquid metal roller-ball pen (LMRP) method [47]. Patterning using LMRP employs a ball pen filled with EGaIn ink. In general, the ball diameter ranges from 200 to 1000 μm, thus the LMRP method is well suited to pattern liquid metal with relatively large line widths.

**Figure 3:**
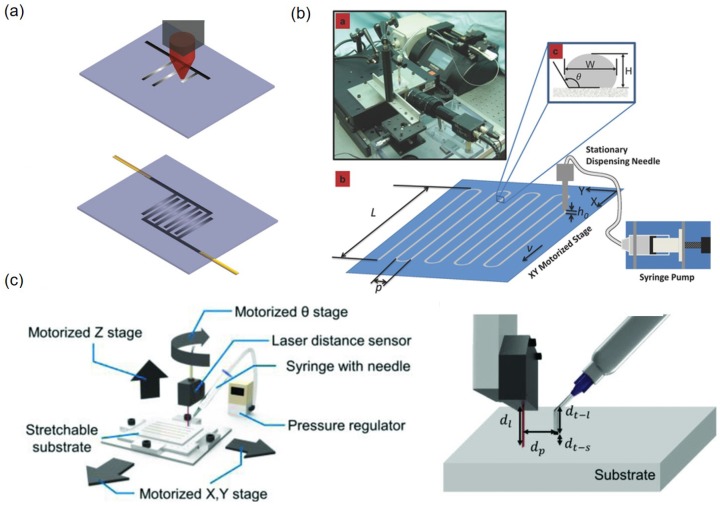
(**a**) Schematic of liquid metal printing using tip that is immersed in liquid metals. After applying liquid metals to the tip, successive droplets are deposited on the substrate and the droplets are merged to form a pattern. Reprinted with permission from [48]. Copyright (2013) American Chemical Society. (**b**) Schematic and photographs of direct printing using motorized stage with syringe pump. The syringe pump discharges the liquid metal and the motorized stage moves to make shape of the pattern. Reproduced with permission from [49]; published by John Wiley and Sons, 2014. (**c**) Schematic of the four-degree-of-freedom liquid metal direct printing. Based on the distance measured from the laser sensor, the distance feedback system allows the distance to be kept constant between the tip and the substrate. Reproduced with permission from [51]; published by John Wiley and Sons, 2019.

It is also possible to print by immersing a microtip in EGaIn and then patterning EGaIn on the substrate point-by-point fashion (Figure 3a) [48]. When small dots are created with their spacings smaller than their diameters, they merge and form patterns. In a more advanced platform, liquid metal printing is performed using a motorized XY-stage and a syringe pump (Figure 3b). While this advanced direct printing [49] is more efficient than the LMRP [47] or the microtip [48] methods, the resulting patterns exhibit undulation due to the pulsation in the syringe pump. To remove the undulation, the syringe pump has been replaced with a pneumatic pressure source and an electronic pressure regulator [50]. It is also important to maintain the distance between the tip and the substrate to obtain a uniform liquid metal pattern. To this end, the distance feedback control is first demonstrated with a motorized XYZ-stage to print linear patterns along one direction on elastomeric substrates [10]. With the aid of the nozzle tip-substrate distance control, EGaIn can be patterned reliably even the patterning substrate is locally irregular. Later, the same concept is extended to enable piece-wise linear patterns with sharp corners on top of various uneven surfaces [51]. This new system is constructed using a motorized XYZ-stage, a motorized rotation stage, and a laser displacement sensor along with an electronic pressure regulator (Figure 3c). The added rotation degree-of-freedom enables continuous EGaIn patterns even when the patterning direction is changed. The rotation stage can steer the laser displacement sensor and make it always precede the patterning nozzle. Therefore, arbitrary piece-wise linear patterns can be printed on inclined or curved substrates.

The width of the directly printed liquid metal patterns depends on the inner diameter of the dispensing nozzle. With the nozzle inner diameter of 83 μm, the minimum width of the EGaIn pattern becomes 44 μm on glass and 34 μm on PDMS, respectively. This is attributed to larger contact angle on PDMS than on glass [49]. This direct printing method can generate various patterns by moving nozzle tip or substrate and the operation is relatively simple. However, the nozzle tip-substrate distance should be well maintained to print continuous liquid metal patterns with high quality.

#### 3.2.2. 3D Printing

**Figure 4:**
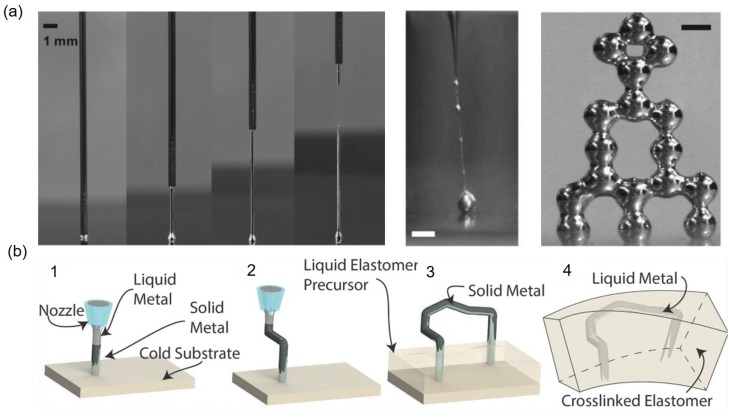
(**a**) Direct writing of a vertical liquid metal wire. Sequential images showing a syringe needle extrudes a liquid metal to form a straight wire by withdrawing the substrate. A tower of droplets made by 3D stacking liquid metal droplets. Scale bars represent 500 μm. Reproduced with permission from [52]; published by John Wiley and Sons, 2013. (**b**) Freezing liquid metals can form a 3D liquid metal structure that can be used as a master mold to make 3D channel. Reproduced with permission from [53]; published by John Wiley and Sons, 2016.

In addition to the direct printing of liquid metal that is basically 2D printing, 3D printing of liquid metal is also feasible since gallium oxides at liquid metal surfaces can support the vertical structure to some degree. The simplest way is to create EGaIn bead at the nozzle tip, make contact with the substrate, and pull it under the same pressure lower than 5 kPa (Figure 4a). In this way, 3D EGaIn structures such as wires, arches, and bridges can be demonstrated along with the motion control of the stage. Individual EGaIn droplets are also used to create 3D structures when they are sequentially stacked on top of each other [52]. To make more stable 3D structures, EGaIn discharged from the nozzle contacts to a cold substrate of which temperature is at 0 °C (Figure 4b). After the EGaIn freezing is initiated at the contact point with the cold substrate, the freezing front moves up and a more stable structure is made compared to 3D structures supported solely by the surface oxide. In this way, high aspect ratio vertical structures with diameter of 250 μm and height of 14 mm can be made. This freezing approach can be combined with the motion control of the XYZ-stage to make more sophisticated 3D structures [53]. Due to the surface oxides around EGaIn, 3D printed liquid metal structures can have diameters same as that of the dispensing nozzle. This is in stark contrast with the 2D direct printing where the pattern width is usually smaller than the nozzle inner diameter. However, it is difficult to print identical structures even under same operating conditions due to high aspect ratio and there are very limited applications.

#### 3.2.3. Inkjet Printing

**Figure 5:**
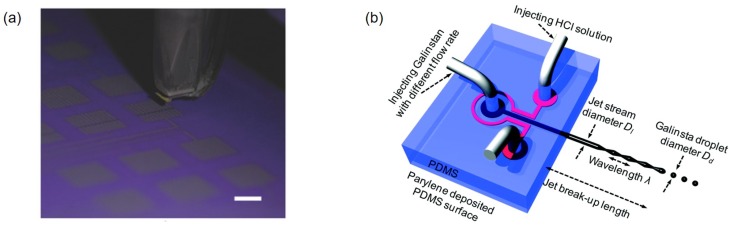
(**a**) Liquid metal patterns are made by using a commercial inkjet printer with liquid metal nanodroplets dispersed ink. Scale bar is 5 mm in length. Reproduced with permission from [55]; published by John Wiley and Sons, 2015. (**b**) An inkjet nozzle made by MEMS fabrication process generating liquid metal microdroplets with the aid of the Rayleigh–Plateau instability. Discharged liquid metal microdroplets can be attached to a substrate by moving XYZ-stage to make desired patterns. Reproduced from Ref. [56] with permission from the Royal Society of Chemistry.

Liquid metal can be also patterned via inkjet printing that is widely used for printed electronics. The inkjet printing is a method of spraying fine ink droplets through many fine nozzles on the printing head [54]. To inkjet print a liquid metal, liquid metal nanodroplets need to be dispersed in a proper solvent first. EGaIn is dispersed in $0.5\times 10^{-3}$ M thiol solution, a stabilizing agent, by sonication for 60 min and then filtered to prepare EGaIn nanofluids. The prepared EGaIn nanofluids exhibit fluidic properties similar to those of the inkjet carrier solvent. Then, EGaIn nanofluids are inkjet printed by ejecting them with ∼65 μm diameter nozzles of a commercial thermal inkjet printer (Figure 5a) [55]. Instead of using a commercial inkjet printer with a 2D array of microsized nozzles, a microfabricated single inkjet nozzle is made with PDMS and used to discharge galinstan microdroplets. Besides the microchannel for galinstan supply and ejection, there is an additional neighboring microchannel for HCl flow (Figure 5b). Since the HCl permeated through the PDMS removes surface oxides of the galinstan inside the microchannel, the injected galinstan can be discharged smoothly through the microsized nozzle and eventually broken up as microdroplets via the Rayleigh–Plateau instability [56,57]. Diameters of the liquid metal droplets discharged from the microfabricated inkjet nozzle can be controlled by the Weber number. Increasing flow rate of the injected liquid metal tends to increase the jetting velocity. Then, the wavelength is decreased and the probability of making small droplets is increased (Figure 5b) [57]. The inkjet printing produces patterns with widths larger than the diameter of ejected liquid metal droplets. For example, a 100 μm wide galinstan pattern can be produced from a 40 μm diameter nozzle [56]. It is possible to make liquid metal patterns of different line widths with the same microfabricated nozzle. However, it is disadvantageous to obtain somewhat non-uniform patterns with undulation that are attributed to non-uniform distribution of generated liquid metal droplets.

#### 3.2.4. Selective Wetting

**Figure 6:**
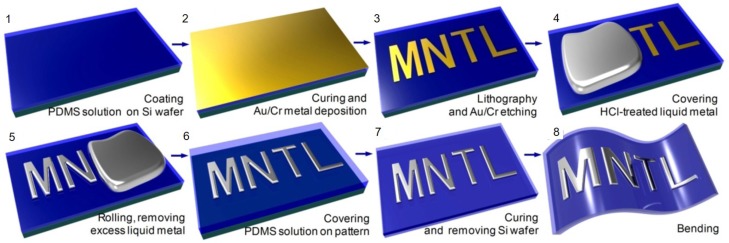
Fabrication process of the liquid metal pattern embedded in PDMS. (1) Spincoating the PDMS solution onto a silicon wafer. (2) PDMS curing, oxygen plasma treatment or parylene deposition, and gold/chromium (100 nm/10 nm) deposition using an electron beam (E-beam) evaporator. (3) Photolithography and gold/chromium wet etching. (4) Dropping HCl-treated liquid metal onto the adhesion patterns. (5) Rolling of the liquid metal over the PDMS substrate and removing the excess liquid metal. (6) Covering the liquid metal pattern with a PDMS solution. (7) PDMS curing and separation of the silicon wafer. (8) Bending test. Reprinted from [58], Copyright (2015), with permission from Elsevier.

Selective wetting is a method of depositing a liquid metal on patterned metal or polymer layers where the liquid metal can be adhered preferentially. In case of metal layers, gold or copper are mainly used. After depositing 10 nm chromium and 100 nm gold layers onto PDMS by using an electron beam evaporator, desired patterns are made by wet etching with a proper etch mask (Figure 6). Then, if galinstan is applied on the PDMS with metals patterned immersed in HCl solution, the galinstan remains selectively adhered to the patterned chromium/gold layer after the PDMS is taken out from the HCl solution [58]. The wetting layer selective to liquid metals can also be patterned with a laser. After sputtering 100 nm copper with 20 nm thick chromium being adhesion layer, the laser ablation removes unwanted parts to create desired metal patterns. This approach easily produces a variety of desired patterns by adjusting the laser path. Once chromium/copper wetting patterns are prepared, EGaIn can selectively adhere to them in NaOH solution [59].

As a polymer wetting layer, polymethacrylates (PMA) glue can be used to increase adhesion between substrate and liquid metal. The adhesion increases due to the hydrogen bond interaction between hydrogen atoms of methyl in the PMA glue and oxygen atoms of the surface oxides on liquid metals. To pattern the PMA glue first, a method similar to the LMRP [47] is used. A ball-point pen filled with the PMA glue directly write PMA patterns on a paper using a motorized stage. With a pen with its diameter of 500 μm, 330 μm wide PMA patterns are made. Then, a hardboard entirely coated with EGaIn and PMA layers by a roller is pressed against the paper with PMA patterns. When the paper with PMA patterns is peeled off, the EGaIn is transferred onto PMA patterns with thickness of ∼50 μm [60,61].

The width of liquid metal patterns is determined by the width of wetting metal or polymer patterns. With the lithographically prepared metal patterns, galinstan patterns down to 3 μm in width can be demonstrated [58]. During the selective wetting, bridging (i.e., unwanted connection) may occur between neighboring wetting patterns when their spacings are small. To prevent this issue, the spacing between adjacent wetting patterns is set to be approximately 1 mm or more [59].

### 3.3. Subtractive Techniques

**Figure 7:**
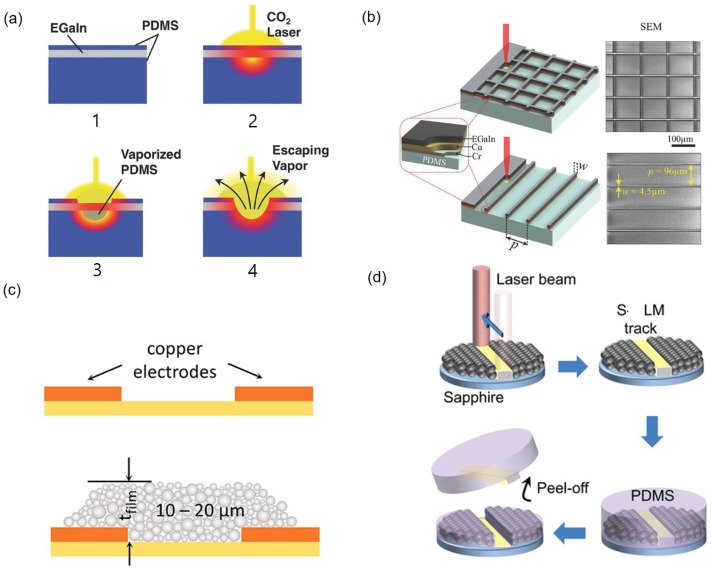
(**a**) Laser ablation creates liquid metal patterns using the liquid metal film between two PDMS layers by removing residual materials. (1) A liquid metal is coated with a thin layer of PDMS to prevent oxidation and limit exposure to debris. (2) Upon exposure to the laser, the sample is locally heated and (3) PDMS from top and bottom layers vaporize. (4) When the pressure difference between the vaporized polymer and atmosphere exceeds the surface tension of the liquid metal film, the vapor will puncture the liquid film and escape. Reproduced with permission from [62]; published by John Wiley and Sons, 2014. (**b**) The schematic and scanning electron microscope (SEM) images of the square grid and parallel line patterns of 4.5 μm wide EGaIn traces; they are patterned by microscale laser ablation of the thin-film architecture (Liquid metal/Copper/Chromium) on a PDMS substrate. Reproduced with permission from [63]; published by John Wiley and Sons, 2018. (**c**) Liquid metal nanoparticles are sintered by using laser on a surface with metal patterns. Reprinted with permission from [64]. Copyright (2018) American Chemical Society. (**d**) Schematic showing sequential procedures for laser writing of liquid metal nanoparticles (LMNPs) into solid–liquid liquid metal patterns on a PDMS substrate. Reproduced with permission from [65]; published by John Wiley and Sons, 2019.

The surface coated with a liquid metal thin film can be patterned using laser ablation, leaving only the necessary parts. By encapsulating the EGaIn film with PDMS, it is possible to generate patterns by selective removal of materials. After spincoating PDMS, EGaIn is deposited as a thin film by using an elastomeric roller, then, it is encapsulated by spincoating another PDMS layer. The laser ablation at wavelength of 10.6 μm vaporizes PDMS due to the local heating. When the vapor pressure is greater than the surface tension of the molten PDMS and the EGaIn, both materials are displaced and removed (Figure 7a) [62]. In another approach, undesired liquid metal area can be selectively removed with the laser ablation on EGaIn film deposited on the copper/chromium (100 nm/20 nm) sputtered PDMS substrate (Figure 7b) [63]. The 10.6 μm wavelength laser produces EGaIn patterns with line widths of 0.1∼1 mm and spacing of 300 μm while the 355 nm wavelength laser produces EGaIn patterns with the minimum width of 4.5 μm and spacing of 100 μm [62,63]. This selective material removal via laser ablation can pattern liquid metal rapidly and change the final shape easily. However, there are several preparation steps before the laser processing and the pattern width strongly depends on the wavelength of the laser.

### 3.4. Nanoparticle Sintering

Liquid metal nanoparticles (LMNPs) can be patterned using thermally induced vaporization and rupture. LMNPs consist of an internal liquid core and an external solid oxide. When LMNPs are heated by pulsed laser irradiation, tension occurs due to thermal expansion of the liquid core and a tail of exudate is formed due to rupture. Oxides that are newly formed on the surface of the exudate fix it to the substrate. During cooling, the LMNPs contract, dimples appear on the surface of LMNPs, and patterns are formed. However, this process occurs when the liquid core temperature is lower than the vaporization temperature. When LMNPs vaporize, liquid cores are radiated, and surface oxides are disrupted. When the temperature is lowered, nanoparticle assemblies can be made and patterned (Figure 7c) [65]. Using the wetting property, sintered EGaIn can be separated from the solid–liquid dual phase. LMNPs are partially sintered on a sapphire to selectively separate the patterns. Subsequently, contacting the PDMS in the solid–liquid dual phase state allows the sintered area to wet the PDMS better so that the desired area can be exclusively transferred (Figure 7d) [65].

Laser sintering can pattern the minimum line width of 200 μm, and the pattern width increases as the laser fluence increases. Since the pattern width also depends on the laser spot, it can be further reduced by configuring a laser with a smaller focal spot [64]. While it is generally difficult to make liquid metal thin films due to its high surface tension, the laser sintering can make liquid metal films with thickness ranging from 10 to 40 μm. In addition, there is an additional advantage that is good adhesion of patterns to the substrate due to the buffer solution where LMNPs are dispersed. To figure out effects of the LMNP diameter on sintered patterns, liquid metal patterns should be generated using LMNPs with a uniform diameter. However, making uniform LMNPs is not a trivial task.

### 3.5. Injection Molding

**Figure 8:**
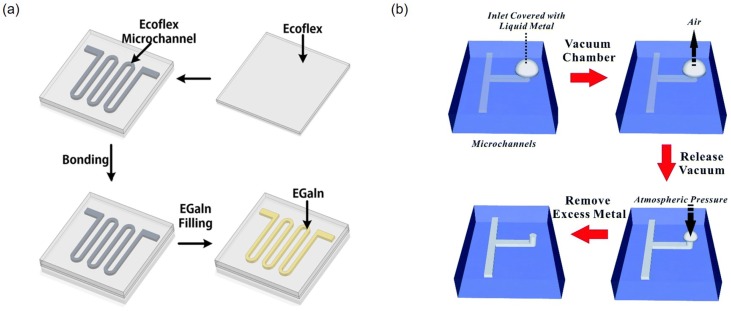
(**a**) Schematic of fabrication process for microchannels made by soft lithography. Injection of a liquid metal in the microchannel makes a liquid metal pattern. Reprinted by permission from Springer Nature: [66], Copyright 2019. (**b**) Schematic showing the sequential process of vacuum filling liquid metal into a T-shaped microfluidic channel with one inlet but no outlets. A liquid metal droplet is dispensed over the inlet. The whole substrate is placed in a vacuum chamber, which removes the air within the microchannel via the gas permeable elastomer. Releasing the vacuum returns the ambient to atmospheric pressure, which pushes the metal into the microchannel. Reproduced from Ref. [67] with permission from the Royal Society of Chemistry.

The injection molding is a method of injecting a liquid metal into microchannels that are made by soft lithography (Figure 8a) [66]. To inject EGaIn into a microchannel that is 20 μm wide and 40 μm tall, a pressure of 89 kPa is required [26]. In general, decreasing the width of the microchannel increases the injection pressure for a liquid metal. If the microchannel is made to have an inlet and no outlet, a negative pressure can be used to introduce a liquid metal. Once EGaIn droplet is placed on the inlet of the microchannel, it is placed in a vacuum chamber for 20 min (Figure 8b). Then, when the vacuum chamber is vented to atmospheric pressure, the EGaIn is filled into the microchannel due to the pressure difference between the microchannel and the chamber. This method can fill microchannels with their widths down to 5 μm [67] and can also work with multiple microchannel branches that have dead ends. Since soft lithography can make microchannels with various widths and heights and provide complicated geometries [68,69], complex liquid metal patterns with a well-controlled cross-section can be fabricated while they are embedded in elastomer microchannels. However, it is difficult to inject liquid metals into very narrow channels due to high surface tension.

## 4. Characterization of Liquid Metal Devices

With the help of electrical and fluidic characteristics, liquid metal devices can be electrically functional under a wide range of stretching, bending, and twisting deformation. Once liquid metal devices are made by any fabrication method, they should be thoroughly characterized prior to be used in practical applications. In this section, major characteristics of liquid metal devices including sensitivity, reliability, and self-healing properties will be discussed.

### 4.1. Sensitivity

**Figure 9:**
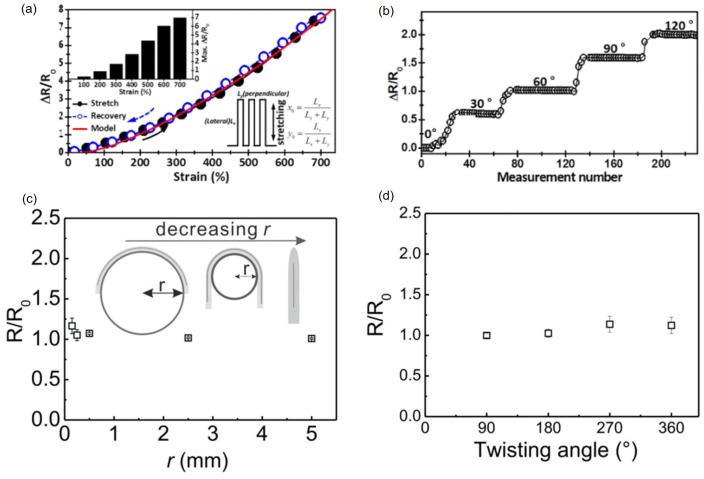
(**a**) Normalized resistance change of the liquid metal strain gauge during the uniaxial stretching test from ϵ = 0 to ϵ = 700%. Measurement is in good agreement with model. © [2015] IEEE. Reprinted, with permission, from [50]. (**b**) Normalized resistance change of the liquid metal strain gauge during the bending test from 0° to 120° © [2015] IEEE. Reprinted, with permission, from [50]. (**c**) Normalized resistance as a function of the bending radius from 0.15 to 5 mm and (**d**) normalized resistance as a function of the twisting angle from 90° to 360° for a resistive liquid metal device encapsulated with PVA at room temperature. Reproduced with permission from [71]; published by John Wiley and Sons, 2019.

To characterize the sensitivity of liquid metal devices, changes in their electrical resistance or capacitance have been measured under the deformation. Mechanical deformation of liquid metal soft sensors can be divided into three types: stretching, bending, and twisting. For resistive liquid metal sensors, either normalized resistance change or normalized resistance are measured under uniaxial strain, bending with a finite angle, folding (i.e., 180° bending) with a finite bending radius, or twisting with a finite angle. A strain gauge of 30 μm wide and 5 mm long EGaIn patterns encapsulated with Ecoflex can be stretched to ∼700% where the normalized resistance change increases to ∼7 (Figure 9a) [50]. However, a strain gauge of 100 μm wide and 40 mm long EGaIn patterns encapsulated with silicone rubber can be stretched to ∼400% [70]. The strain limit actually results from the encapsulating materials since liquid metals deform with more freedom. In addition, stretchable sensors made of EGaIn have a gauge factor (GF) of 4.95 at 550% strain [66]. For bending tests, a liquid metal strain gauge is attached to the forefinger joint and normalized resistance change is measured at different bending angles. The normalized resistance change increases as the bending angle increases. The maximum value is 2 at 120°, which is equivalent to ∼350% of the uniaxial strain (Figure 9b) [50].

A 340 μm wide and 4 cm long galinstan pattern encapsulated with poly(vinyl alcohol) (PVA) is bent with the bending radius ranging from 0.15 to 5 mm (Figure 9c). With the large bending radius of 2.5 or 5 mm, there is almost no change in the normalized resistance. With the bending radius below 1 mm, the normalized resistance increases due to the high local strain. At 0.15 mm, the smallest bending radius tested, the normalized resistance becomes the maximum 1.17 [71]. Using the same device used in Figure 9c, the normalized resistance is measured with twisting angle of 90°, 180°, 270°, and 360° (Figure 9d). The normalized resistance does not change much at 90° and 180°. However, the normalized resistance increases by 10% at 270° or 360° [71]. While the resistance change of the sensor according to the strain (Figure 9a) and bending angle (Figure 9b) is relatively large, the resistance is almost constant when the bending radius (Figure 9c) and twisting angle (Figure 9d) are changed.

**Figure 10:**
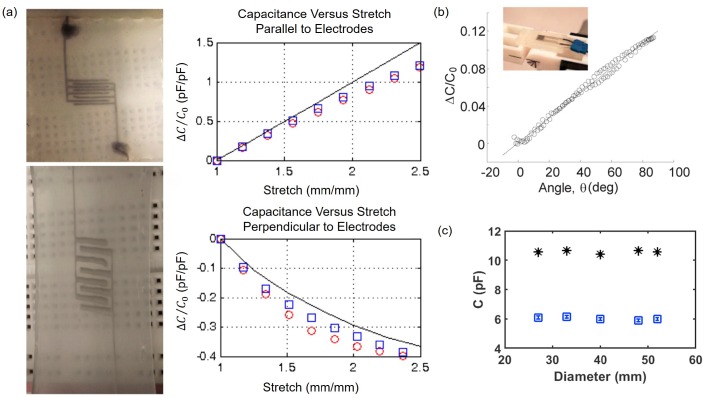
(**a**) Photographs of an interdigitated liquid metal capacitor stretched parallel (top left) and perpendicular (bottom left) to electrodes after uniaxial stretching of 250%. Normalized capacitance changes in the interdigitated capacitor as a function of uniaxial stretch parallel (top right) or perpendicular (bottom right) to electrodes. In each case, experiments (represented by red and blue markers) are conducted twice and black solid lines are theoretical predictions. Reprinted with permission from [48]. Copyright (2013) American Chemical Society. (**b**) Normalized capacitance changes of a parallel-plate liquid metal capacitor as a function of the bending angle. Reprinted with permission from [72]. Copyright (2015) American Chemical Society. (**c**) Capacitance as a function of the bending diameter. Two types of interdigitated liquid metal capacitors with their total width of 27 (black) or 57 mm (blue) are used. Reprinted from [73], Copyright (2015), with permission from Elsevier.

Interdigitated capacitors made of 340 μm wide EGaIn patterns encapsulated with silicone elastomer are stretched in parallel or perpendicular to electrodes (Figure 10a). As λ, the ratio of extended length to the initial length due to tension, increases, normalized capacitance change increases in the direction parallel to electrodes but decreases in the direction perpendicular to electrodes. The normalized capacitance change in the direction parallel to electrodes is more sensitive than that in the direction perpendicular to electrodes [48]. In addition, normalized capacitance change of parallel-plate capacitor made of EGaIn pattern and PDMS encapsulation is measured and plotted as a function of the bending angle (Figure 10b). The normalized capacitance change becomes the maximum of 0.12 when the bending angle is increased from 0° to 100° [72]. Interestingly, this is one order of magnitude smaller than the normalized resistance change of the liquid metal strain gauge that undergoes the same bending deformation [50]. Using two type of interdigitated capacitors made of EGaIn patterns encapsulated with PDMS with small (total width and length of 27 and 40 mm, respectively) or large (total width and length of 57 and 40 mm, respectively) fingers, capacitance change is measured and plotted as a function of the bending diameter. The measured capacitance is nearly constant regardless of the bending diameter ranging from 28 to 50 mm (Figure 10c) [73]. When 30 μm wide, 31 μm tall, and 200 μm long galinstan patterns encapsulated by PDMS are twisted to 30° or 60°, difference of capacitance compared to the value at a relaxed state are only ∼4%, so there is negligible difference in the capacitance due to twisting [74]. Negligible effect of the bending radius and twisting angle on sensor output is common for both resistive and capacitive liquid metal sensors.

### 4.2. Reliability

**Figure 11:**
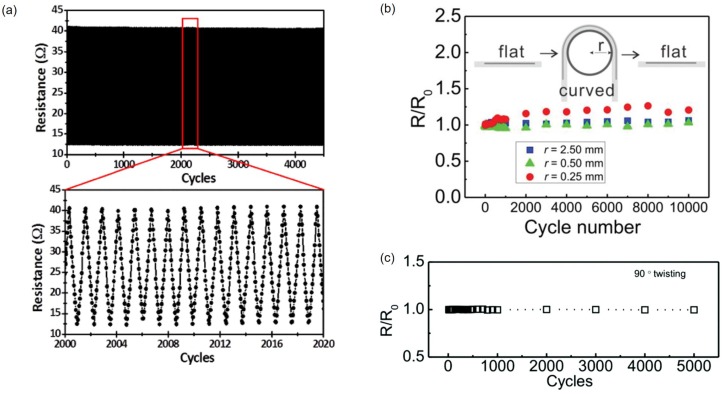
(**a**) Resistance measurement of the liquid metal strain gauge for 4500 cycles at ϵ = 350%. The zoom-in shows ∼20 cycles. There is no noticeable degradation. © [2015] IEEE. Reprinted, with permission, from [50]. (**b**) Normalized resistance measurement of patterned galinstan resistors for 10,000 bending cycles at bending radii of 0.25, 0.5, and 2.5 mm. Reproduced with permission from [71]; published by John Wiley and Sons, 2019. (**c**) Normalized resistance measurement of patterned EGaIn resistors for 10,000 twisting cycles at twisting angle of 90°. Reproduced from Ref. [44] with permission from the Royal Society of Chemistry.

To use liquid metal sensor devices for extended period of time, reliability should be well characterized. When the 4500 continuous cycles are performed for 350% uniaxial strain on a strain gauge made of 30 μm wide and 5 mm long EGaIn patterns encapsulated by Ecoflex, it can be driven without noticeable degradation (Figure 11a) [50]. At 50% strain, normalized resistance of a strain gauge which is made by 900 μm wide galinstan patterns encapsulated with silicone rubber is measured with the largest repeated cycles of 10,000 [75]. In this case, however, degradation occurs at both the maximum and minimum values of normalized resistance [76]. Unlike strains, there is almost no change in resistance according to change of bending radius. However, in repeated tests, normalized resistance tends to increase with decreasing bending radius. After bending 340 μm wide and 4 cm long galinstan patterns encapsulated by PVA are bent to radius of 0.25, 0.5, or 2.5 mm, normalized resistance is nearly unchanged with the bending radius equal to or greater than 0.5 mm during 10,000 cycles. However, at 0.25 mm bending radius, the normalized resistance increases to 1.25 after 10,000 cycles (Figure 11b) [71]. In addition, twisting has a same normalized resistance in repeated tests. EGaIn pattern of 289 μm width and 9.71 mm length encapsulated by PDMS shows normalized resistance as 1 even after 5000 repeated tests at 90° twisting angle (Figure 11c) [44]. Even for repeated deformations beyond 10,000 cycles, the effect of surface oxide would be saturated thus would not change electrical characteristics of liquid metal devices.

**Figure 12:**
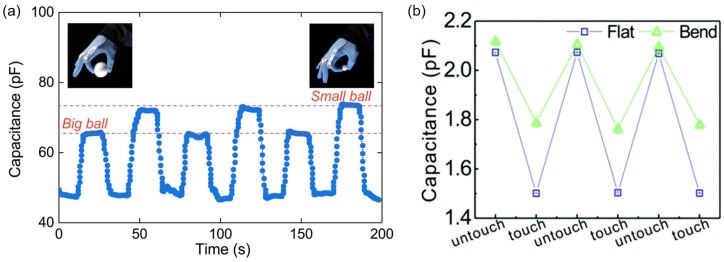
(**a**) Capacitance change of a parallel-plate liquid metal capacitor measured for 200 s. When big and small balls are picked up alternatingly, the capacitor experiences two different bending angles thus shows two constant levels during the repeated test. Reprinted with permission from [78]. Copyright (2019) American Chemical Society. (**b**) Capacitance measurement of a fork-shaped EGaIn electrode in untouched and touched states during three repeated cycles. If the capacitor is flat in the repetition cycle, the capacitance difference is larger than that for the bend. Reproduced from Ref. [44] with permission from the Royal Society of Chemistry.

To check the reliability of capacitive liquid metal sensors, capacitance should be measured repeatedly for cycles equivalent to resistive liquid metal sensors. However, much fewer cycles have been demonstrated and reported. Four repeated tensile tests are performed for interdigitated capacitors made of 400 μm wide and 500 μm tall galinstan patterns with 17% increase in the direction parallel to electrodes. Two samples show the same behavior in loading and unloading with standard deviation less than 0.002 [77]. To check the reliability with the bending, parallel-plate liquid metal capacitors attached to a glove are bent at different angles while holding Styrofoam balls of 47 mm and 19 mm diameter, respectively. When big and small balls are picked up alternatingly, the capacitor experiences two different bending angles thus show two constant levels, 65 and 72 pF for big and small balls, respectively, during the repeated test (Figure 12a) [78]. In addition, capacitance readings with 500 μm wide fork-shaped EGaIn electrodes are used to sense touch or untouch events. In three iteration cycles, flat or bent devices exhibit capacitance differences of 0.6 and 0.3 pF, respectively, and they have the same maximum and minimum values (Figure 12b). The reason for having a larger capacitance in the flat state than that in the bent state is due to changes in the contact area [44].

### 4.3. Self-Healing

A unique characteristic of the liquid metal devices is self-healing that devices self-repair damages. Liquid metals make thin oxide layers on interfacial areas so quickly that new oxide layers can form on surfaces of liquid metals even if they are under deformation. When liquid metal patterns are reconnected after a damage, they tend to merge due to high surface tension. In addition, oxides are newly created. Therefore, a self-healing process is favorable [79].

**Figure 13:**
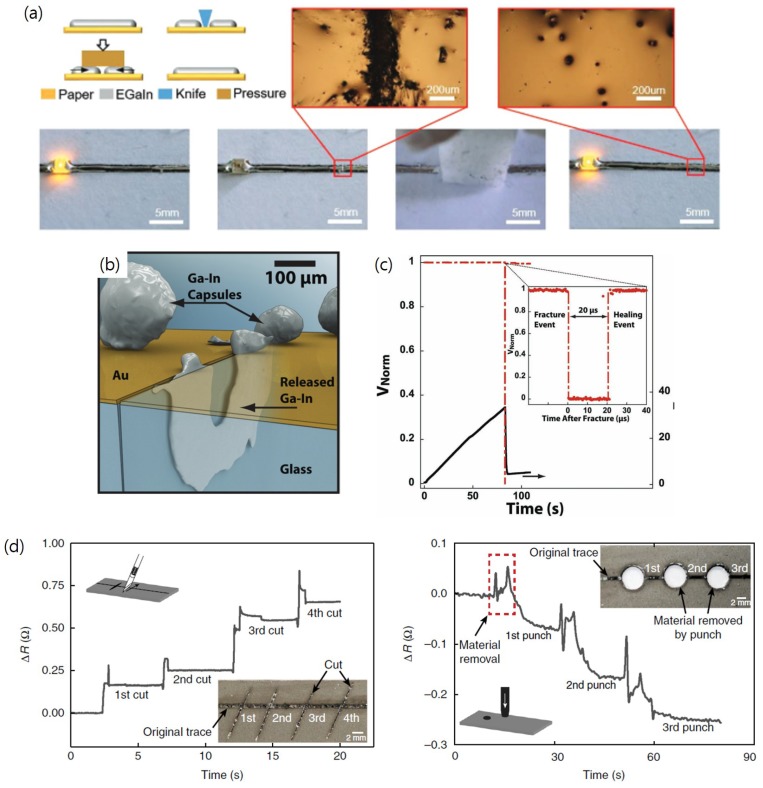
(**a**) Self-healing procedures and corresponding photographs of a liquid metal circuit. The liquid metal circuit damaged by knife is recovered by applying pressure. Reproduced with permission from [60]; published by John Wiley and Sons, 2019. (**b**) Illustration showing the crack in a thin gold film on glass repaired by released EGaIn capsules. (**c**) Normalized bridge voltage (red) and force (black) measurements recorded during the four-point bending test of a self-healing specimen. Reproduced with permission from [80]; published by John Wiley and Sons, 2019. (**d**) Measured resistance changes of liquid metal patterns damaged by razor blade cutting (high aspect ratio damage, left) and by hole punching (low aspect ratio damage, right). Reprinted by permission from Springer Nature: [81], Copyright 2018.

Recently, the self-healing ability of liquid metal devices are experimentally investigated. EGaIn patterns are first cut off by knife and external pressure is applied to the damaged region (Figure 13a) [60]. To check the circuit connection and evaluate the self-healing, an LED is inserted within EGaIn patterns. Both micrographs and photographs in Figure 13a show that EGaIn fills the separated gap and repairs the damage after the pressurization. In another study, the self-healing property inspires development of other liquid metal embedded devices. Liquid metal microcapsules, microdroplets enclosed by surface oxide shell that are dispersed on top of gold thin films with cracks, have proven the ability of filling cracks (Figure 13b) [80]. 3 μm EGaIn microcapsules are ruptured to repair cracks in the circuit. The four-point bending test is also performed to characterize the self-healing event using a Wheatstone bridge with or without one bridge arm being the specimen. While the bending force is increased linearly until a crack propagates through the circuit, the normalized bridge voltage is monitored and recorded. With the self-healing specimen, the normalized bridge voltage is quickly restored to ∼99% of the original value within 20 μs (Figure 13c). However, no recovery is observed without the self-healing specimen. By making reliable numbers of interfaces between liquid metals and other solid conductive materials, devices can have consistent thermal or electrical conductivities while cracks propagate through solid conductive materials upon extreme deformation.

The autonomous self-healing elastomer aforementioned maximizes the self-healing property of liquid metals for the sensor reliability. Markvicka et al. [81] developed liquid metal embedded elastomers and check their self-healing property. The liquid metal embedded elastomers are given high aspect ratio damages by cutting with a razor blade or low aspect ratio damages by punching with a hole punch (Figure 13d). Small increase in resistance is observed for the liquid metal embedded elastomer damaged by the razor blade (<0.75 Ω in total or <0.2 Ω per each damage). However, small decrease in resistance is observed for the liquid metal embedded elastomer damaged by the hole punch (<0.3 Ω in total or <0.1 Ω per each damage). The decrease in resistance for the punching damage is attributed to two branches for current flow (i.e., upper and lower semi-circles) made upon punching.

## 5. Applications

The most important feature of liquid metal devices is their superior deformability compared to devices made with solid-state conductive materials. To take advantage of this unique and promising feature, liquid metals can be mainly used in two ways. First, they can be used to interconnect solid-state electronic parts and offer mechanical flexibility to the connected system. Second, they can become a standalone sensor of which resistance, capacitance, or inductance can change upon exposure to a proper stimulus. In this section, applications of liquid metals including interconnections, resistive and capacitive sensors will be discussed.

### 5.1. Interconnections

**Figure 14:**
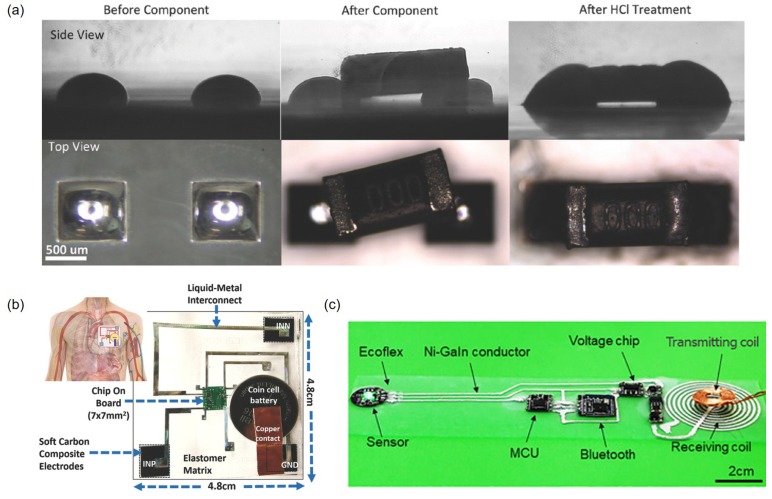
(**a**) Liquid metal is used as a solder paste for surface mount passive components. The components misaligned on liquid metal pads are aligned by HCl treatment. Reproduced with permission from [59]; published by John Wiley and Sons, 2018. (**b**) Liquid metal interconnects are used for the stretchable electrocardiogram patch. Reproduced with permission from [84]; published by John Wiley and Sons, 2019. (**c**) An overview of the pulse measurement circuit. Solid-state electronic parts are connected with liquid metal interconnections. Reproduced with permission from [82]; published by John Wiley and Sons, 2018.

One simple way to make a liquid metal interconnection is to directly contact liquid metal patterns with solid-state conductors or components. A wire or a microcontroller unit (MCU) can be placed on the liquid metal layer or soaked if the layer is thick [82,83]. The HCl vapor treatment with a solid-state electronic component on top of EGaIn pads removes surface oxides and enables self-alignment due to the high surface tension of the liquid metal (Figure 14a) [59]. In the aforementioned method, encapsulation is performed after all necessary solid parts are connected. However, there is a possibility that connections easily fail due to the somewhat loose link between liquid metals and solid-state electronic components especially when there is external impact. To solve this issue, a more reliable contact is made by connecting the solid pad of the chip with the end of the microchannel filled with galinstan [84] or connecting solid-state components with substrate grooves that are filled with a EGaIn to fix contacts [85]. Liquid metal interconnections have been mainly applied to wearable devices that are attached to human skin, such as stretchable electrocardiogram patches (SEPs) and healthcare monitors for pulse measurements (Figure 14b,c) [82,83,84]. It is expected that liquid metal interconnections will be applied to other wearable device applications. However, the interconnecting area where liquid metal and solid conductor meet is still vulnerable to failure. To interconnect liquid metal patterns and solid conductors, solid conductors are placed into [82] or onto [51] liquid metal patterns or a liquid metal is directly patterned on solid conductors that are typically thin films [86]. Since there is no interface conductive material with intermediate mechanical properties between liquid and solid conductors such as gel, the interconnecting area between them tends to fail if it is strained beyond a critical limit.

### 5.2. Sensors

#### 5.2.1. Resistive Sensors

**Figure 15:**
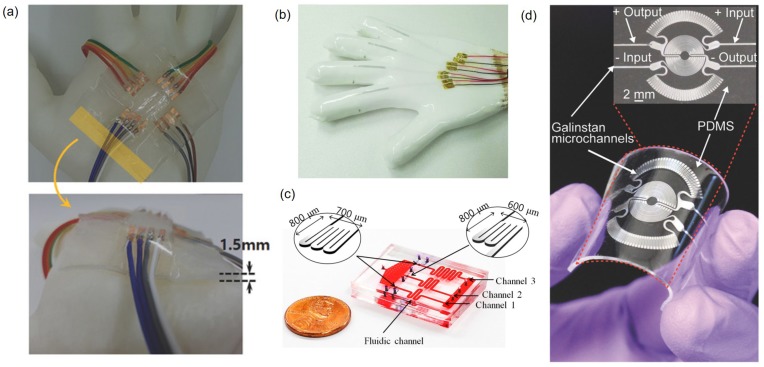
(**a**) Pictures of the 2D tactile sensor exhibiting 4 horizontal and 4 vertical EGaIn lines, thus 16 intersecting points. Reproduced with permission from [10]; published by John Wiley and Sons, 2018. (**b**) Liquid metal patterned glove that captures hand or finger gestures. Reproduced with permission from [51]; published by John Wiley and Sons, 2019. (**c**) Resistive liquid metal pressure sensor which measures the pressure in microfluidic channels. Reproduced with permission from [93]; published by MDPI, 2015. (**d**) Wearable liquid metal pressure sensor that monitors the heart rate to detect the BPM. Reproduced with permission from [94]; published by John Wiley and Sons, 2017.

In deformable electronics, the deformation of sensors causes the length and cross-sectional area of the liquid metal patterns to change [87]. Therefore, devices using liquid metal patterns can be used to measure strain and detect motions by resistance changes according to the deformations. The resistive strain sensor is one of the most widely used applications of soft sensors made with liquid metals. Liquid metal-based strain sensors can be applied to human body joints experiencing extreme deformation such as ankle, hip, and knee of which typical strain levels are 130∼150, 270∼380, and 290∼480%, respectively [88]. EGaIn strain sensors made to measure joint angles can be stretched up to ∼400% longer than their original length (i.e., strain of ∼500%) [70]. Multiple liquid metal strain gauges are configured as a 2D orthogonal mesh format on a plane to become a tactile sensor (Figure 15a) [10]. This tactile sensor exhibits the dynamic range of 0∼90 kPa and is also well suited for electronic skin applications since its resolution is better than 5 kPa that has been previously reported as a value required to recognize a gentle touch on human skin [89].

In addition, detection of complicated hand motions or gestures is possible if multiple liquid metal strain sensors are directly integrated on each finger on a glove by direct printing of EGaIn (Figure 15b) [51,86]. Once movement of each finger measured with an individual strain sensor is combined, a variety of hand gestures can be differentiated thus detected. Similar wearable gloves can be made with silver nanoparticle thin films [90], silver nanowire reinforced conductive fibers [91], or carbon nanotube films [92]. However, they need to be used to make individual strain sensors that would be attached on a glove later. By contrast, in case of liquid metals, strain sensors can be directly printed thus seamlessly integrated on a glove.

Pressure can be measured by the resistance change caused by deformation in cross-sectional area of liquid metal patterns. For example, pressure changes in a microfluidic system can be measured with an integrated liquid metal resistive sensor. The device consists of a thin PDMS membrane between fluidic channels at the bottom layer and the pressure sensor channel filled with galinstan at the top layer (Figure 15c). When inlet and outlet pressures of fluidic channels are identical, there is no change in resistance of the liquid metal sensor. However, when the inlet pressure is larger than the outlet pressure, the channel cross-sectional area decreases and the resistance increases. Once calibrated, the differential pressure between inlet and outlet can be measured [93].

On a similar principle, liquid metals can also be used to make diaphragm pressure sensors. For example, diaphragm pressure sensors made with galinstan enable the heart rate monitoring (Figure 15d), one of the most popular applications in wearable electronics, by measuring the resistance. For a person exercising on a cycling ergometer, data from the galinstan diaphragm pressure sensor show good agreement with those from a commercial heart rate monitoring system [94].

#### 5.2.2. Capacitive Sensors

**Figure 16:**
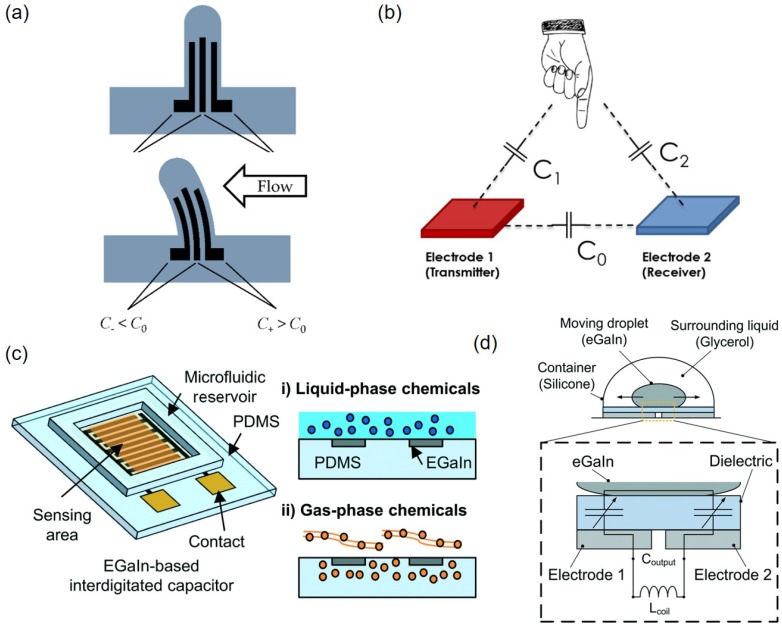
(**a**) Capacitance of a liquid metal sensor changes depending on the flow rate of medium. Reproduced with permission from [95]; published by MDPI, 2019. (**b**) Capacitance change due to proximity can be used to detect the distance differences. Reproduced with permission from [96]; published by MDPI, 2019. (**c**) Schematic of an EGaIn-based interdigitated capacitor with a microfluidic reservoir for sensing liquid- or gas-phase volatile organic compounds. Reproduced from Ref. [99] with permission from the Royal Society of Chemistry. (**d**) An inertial sensor using a liquid metal droplet can detect the motion by measuring capacitance change. Reproduced from Ref. [102] with permission from the Royal Society of Chemistry.

Liquid metals can also be used to make a capacitor encapsulated within soft materials and employed as a sensor to detect a stimulus to induce the capacitance change. Liquid metal-based capacitive sensors have been used to measure flow rate (Figure 16a) [95] and pressure [96,97]. In addition, the proximity effect around a liquid metal-based capacitive sensor can be used to detect the difference in distances from two electrodes (one is transmitter and the other is receiver) (Figure 16b) [98]. Interdigitated capacitive sensors made with a liquid metal can measure liquid-phase or gas-phase volatile organic compounds (VOCs). Especially for liquid-phase analytes, a microfluidic reservoir of 136.5 μL is integrated on top of interdigitated EGaIn electrodes (Figure 16c). For liquid-phase VOCs, the relative permittivity of the chemical under test is an important factor resulting in a linear change in capacitance. Since the sensor also shows a linear change in capacitance depending on the volumetric mixing ratio of deionized water and target chemical, the change in composition of liquid analytes can be detected. For gas-phase VOCs, the PDMS substrate where interdigitated EGaIn electrodes are engraved is used as a sensing film with the aid of gas diffusion, so that there is no need to integrate the sample reservoir [99]. As the normalized capacitance change increases with the concentration of gaseous analytes, gas-phase VOC concentrations can be measured.

Moreover aforementioned sensing mechanisms, the capacitance change due to the change of overlapping areas can be employed. Application or release of load or pressure can be detected using the capacitance change depending on the change of overlapping area between a liquid metal droplet and an underlying electrode [100,101]. Similarly, a liquid metal droplet is used to recognize the gesture by the capacitance change (Figure 16d). When the EGaIn droplet inside the sensor moves, the overlapping area with adjacent electrodes changes that in turn induces the output capacitance change. When a liquid metal capacitor is combined with a liquid metal inductor, motion detection that induces capacitance change can be measured by monitoring the resonant frequency of the LC network [102].

The capacitance also varies with the dielectric constant that may be affected by the volume percent of liquid metal particles dispersed in soft dielectric materials. EGaIn micro- and nanoparticles are dispersed in Ecoflex by high-shear mixing or sonication. The resulting liquid metal-elastomer composite can be used as a dielectric layer between electrodes. Relative permittivity of the composite increases with increasing the volume ratio of EGaIn particles. For example, the EGaIn particles-Ecoflex composite with the particle volume ratio of 80% exhibits the relative permittivity that is ∼27 times larger than that of the bare Ecoflex without EGaIn particles at room temperature [78]. By manipulating the particle volume percent, relatively large capacitance can be achieved while maintaining the same sensor size.

## 6. Challenges and Future Works

Since liquid metal patterning is enabled with the help of the surface oxide, it is not possible to remove the oxide and we need to use liquid metal devices in its presence. The surface oxide is known to be on the order of a few nanometers; however, the measurement accuracy for its thickness is still questionable. Although the thickness or volume of the oxide is considerably smaller than the height or volume of the enclosed liquid metal, the effect of the thin oxide on electrical or mechanical properties of the entire liquid metal pattern is not fully understood. Therefore, a thorough study to measure the oxide thickness with high accuracy should be conducted and theoretical and experimental studies should be performed on the effect of the oxide presence on other relevant properties and the behavior of the entire device.

While a variety of patterning methods for liquid metals have been developed and demonstrated to date, none of them are yet high-throughput. To expedite the development of promising liquid metal devices and make them even commercially viable, it is necessary to push the patterning techniques to the next level. For example, the contact printing based on elastomeric stamps [103] can be upgraded to roll-to-roll-based continuous printing and the dispensing nozzle-based direct printing can be significantly improved by configuring multiple dispensing nozzles.

Except liquid metal devices with wireless data transmission, liquid metal patterns should be connected to outside world via solid connectors. In addition, solid-state passive or active components can be placed on liquid metal patterns. Although connection areas between liquid metals and solid components or connectors are placed on regions under negligible or at least minimum deformation, they are the most vulnerable spots due to the mechanical mismatch. Therefore, it is urgent to develop more reliable packaging to connect liquid metal patterns and solid parts. A possible solution is to introduce an interface material that exhibits decent electrical conductivity as well as mechanical compliance between liquid metals and solid parts.

Another interesting direction to pursue with liquid metals is to make composites with other materials to integrate additional functionalities. For example, liquid metal composites can be made by dispersing liquid metal droplets into soft polymers [78] or forming polymeric ligand encapsulation chemically in liquid metals to produce functional polymers with high electrical and thermal conductivities [104]. In addition, nickel or iron particles that are magnetic at or near room temperature can be dispersed in liquid metals [61,105]. Such magnetic particles laden liquid metals can offer both electrical and magnetic properties while accommodating large deformation.

## 7. Conclusions

This paper reviews fundamentals, fabrication, characterization and applications of liquid metal devices with a specific focus on soft and deformable sensors. Stretchability, flexibility and conductivity of liquid metals make them well suited for applications requiring electrical functionalities under large mechanical deformation. Fortunately, a thin solid oxide layer on liquid metal surfaces enables liquid metal patterning on various substrates, leading to a variety of applications including interconnects and sensors. Besides direct printing of liquid metals, various established microfabrication techniques and laser processing can be applied for liquid metals to realize more sophisticated devices. Even though there are still many challenges to overcome, liquid metals are certainly rising stars in the era of wearable electronics and soft robotics.
